# Red Blood Cell Distribution Width Is Not a Predictor of Hospital Mortality in Elderly and Nonelderly COVID-19-Infected Patients: A Prospective Study at a Brazilian Quaternary University Hospital

**DOI:** 10.1155/cjid/2118702

**Published:** 2025-09-15

**Authors:** Helena Duani, Máderson Alvares de Souza Cabral, Carla Jorge Machado, Thalyta Nogueira Fonseca, Milena Soriano Marcolino, Vandack Alencar Nobre, Cecilia Gómez Ravetti, Paula Frizera Vassallo, Unaí Tupinambás

**Affiliations:** ^1^Programa de Pós-Graduação em Ciências da Saúde: Infectologia e Medicina Tropical, Faculdade de Medicina, Universidade Federal de Minas Gerais, Belo Horizonte, Brazil; ^2^Serviço de Doenças Infecciosas e Parasitárias, Hospital das Clínicas, Universidade Federal de Minas Gerais, Belo Horizonte, Brazil; ^3^Departamento de Clínica Médica, Faculdade de Medicina, Universidade Federal de Minas Gerais, Belo Horizonte, Brazil; ^4^Programa de Pós-graduação em Promoção de Saúde e Prevenção da Violência, Faculdade de Medicina, Universidade Federal de Minas Gerais, Belo Horizonte, Brazil; ^5^Unidade de Terapia Intensiva Adulto, Hospital das Clínicas, Universidade Federal de Minas Gerais, Belo Horizonte, Brazil

**Keywords:** biomarkers, COVID-19, hemogram, mortality, RDW, SARS-CoV-2

## Abstract

**Objective:** To investigate if red blood cell distribution width (RDW) is a risk factor for hospital mortality in patients admitted to a public university hospital in Belo Horizonte, Minas Gerais, Brazil.

**Methods:** This observational prospective study included patients over 16 years who had been hospitalized for COVID-19 between May and October 2020. A descriptive and time-to-death analysis was performed using the Cox proportional hazards model.

**Results:** Of the 161 patients included, 39 (24.2%) died during hospitalization. A total of 2227 blood counts were performed, an average of 13.8 tests per patient (standard deviation, SD 2.9). Upon admission, the RDW was normal (11.5% to 14.6%) in 115 patients (71.4%), elevated in 45 (28%), and low in 1 (0.6%). The mean RDW value at admission was 14.5 (SD 2.4), which falls within the normal reference range. Of the patients with normal RDW at admission, 82 (71.3%) maintained normal levels throughout their stay, while 33 (28.7%) showed increased RDW levels over time. Among those with elevated RDW at admission, 40 (88.9%) remained elevated, while 5 (11.1%) returned to normal levels. There was no significant difference in the mean RDW value at admission between survivors and nonsurvivors (14.4 [SD 2.4] for survivors vs. 14.9 [SD 2.4] for nonsurvivors; *p*=0.2081). The risk for mortality in the group with high RDW upon admission was higher than in the group with normal RDW, but without statistical significance (31.1% vs. 21.7%; RR = 1.43; *p*=0.413). When performing a multivariate analysis, the following continue to be risk factors for lower survival: age > 70 years, HR 4.8 (95% CI: 2.3; 10.3), *p* < 0.001; white race, HR 3.2 (95% CI: 1.2; 8.61), *p*=0.018; and need for invasive MV, HR 3.8 (1.7; 8.7), *p*=0.001. The presence of a chest X-ray suggestive of COVID-19, HR 3.5 (95% CI: 1.0; 11.5), *p*=0.044, also appears to be a risk factor in this analysis.

**Conclusion:** Alterations on RDW values on admission were not associated with higher mortality, and further increases in RDW during hospitalization were not linked to a higher risk of mortality across all age groups. Our findings suggest that while RDW may indicate disease severity, it may not serve as a reliable independent predictor of mortality when other factors are accounted for in this cohort.

## 1. Introduction

In December 2019, an outbreak of atypical viral pneumonia cases emerged in Wuhan, China, marking the beginning of what would become known as coronavirus disease 2019 (COVID-19). The etiologic agent was quickly identified as a novel beta-coronavirus, designated as severe acute respiratory syndrome coronavirus 2 (SARS-CoV-2). This highly transmissible and pathogenic virus rapidly spread globally, prompting the World Health Organization to declare a pandemic in March 2020 [1].

The clinical manifestation of COVID-19 encompasses a broad spectrum, ranging from asymptomatic infection to critical illness requiring intensive care support. A narrative review of international cohort studies reported asymptomatic infection rates varying from 6.3% among nursing facility residents in the United States to 96% in incarcerated individuals [2]. Data from a Chinese cohort with over 44,000 COVID-19-infected patients from the early pandemic have shown that 81% developed mild disease, 14% severe, and 5% critical illness [3]. Risk stratification analyses have identified several factors associated with increased disease severity and mortality. Advanced age represents a significant independent risk factor, with individuals over 65 years experiencing disproportionately higher rates of severe outcomes. A meta-analysis of 42 studies including 423,117 patients estimated pooled odds ratios (pOR) and hazard ratios (pHR) for older age at 2.61 (95% CI 1.75–3.47) and 1.31 (95% CI 1.11–1.51), respectively (Doe et al., 2021). Male sex (pOR = 1.45; 95% CI 1.41–1.51) and current smoking status (pOR = 1.42; 95% CI 1.01–1.83) were also significantly associated with higher mortality risk [4].

Additionally, comorbidities such as cardiovascular disease (pOR = 1.83; 95% CI 1.50–2.17), hypertension (pOR = 1.57; 95% CI 1.27–1.87; pHR = 1.18; 95% CI 1.01–2.07), chronic obstructive respiratory disease (pOR = 1.58; 95% CI 1.08–2.02; pHR = 1.71; 95% CI 1.01–1.40), diabetes mellitus (pHR = 1.52; 95% CI 1.36–1.69), cancer (pHR = 1.33; 95% CI 1.09–1.56), and obesity (pOR = 1.34; 95%CI = 1.17–1.52; pHR = 1.50; 95%CI = 1.26–1.75) substantially elevate the risk of adverse outcomes [4].

In the pursuit of improved prognostication and patient risk stratification, researchers have identified several circulating biomarkers that correlate with disease severity and clinical outcomes. Elevated D-dimer levels, lymphocytopenia [5, 6], and increased red blood cell distribution width (RDW) have emerged as potential predictors of adverse outcomes. Of particular interest, studies have shown that patients presenting with elevated RDW upon hospital admission, as well as those experiencing an increase in RDW during hospitalization, exhibit a significantly higher risk of mortality [7].

This study examines the role of RDW in predicting outcomes in COVID-19-infected patients. We focused on both the initial RDW values at admission and their changes during the hospital stay. Our goal was to analyze the link between RDW levels and in-hospital mortality in patients treated at a Brazilian public university hospital. By looking at both baseline RDW values and their trends over time, we aimed to determine whether these parameters could help predict poor outcomes in hospitalized COVID-19-infected patients.

## 2. Materials and Methods

All patients aged 16 years or older, admitted between May 1 and October 31, 2020, to the University Hospital at the Universidade Federal de Minas Gerais (HC-UFMG) in Belo Horizonte, Brazil, were included. Patients presented with SARS or other clinical symptoms that raised suspicion of COVID-19 within the first three weeks of their hospitalization. Patients hospitalized for other reasons who subsequently contracted COVID-19 during their stay were excluded from the study. Belo Horizonte, the capital of southeastern Brazil, has a population exceeding 2.5 million, with over 80,000 residents aged between 70 and 74, reflecting the city's considerable elderly population (source: https://cidades.ibge.gov.br/brasil/mg/belo-horizonte/panorama, accessed on July 11, 2024).

The HC-UFMG is a tertiary care facility with a capacity exceeding 400 inpatient beds, serving as a regional referral center for complex medical interventions, including hematopoietic stem cell and solid organ transplantation and oncological treatments. Following the emergence of SARS-CoV-2, the institution was integrated into the Brazilian Unified Health System's (SUS) network of COVID-19 treatment centers, specifically serving the metropolitan region of Belo Horizonte. The study protocol implemented daily prospective data collection through systematic surveillance conducted by the research team. Data acquisition was performed via the institution's electronic health records system, incorporating clinical documentation, radiological findings, and laboratory results.

Data were collected through a questionnaire administered during the study's follow-up to gather relevant variables, which were compiled into a database. The race/ethnicity variable was self-reported by the participants.

A confirmed COVID-19 case was defined as a patient with a positive real-time polymerase chain reaction (RT-PCR) test for the detection of SARS-CoV-2, using either tracheal aspirate or naso-oropharyngeal swabs. Cases that were only based on clinical suspicion and radiological findings, without laboratory confirmation, were excluded from the study.

Immunosuppression was defined as the presence of cancer, an immunosuppressive hematologic condition, primary or acquired immunodeficiency, or any relevant clinical condition requiring treatment with immunosuppressants or high doses of corticosteroids. The first chest X-ray or computed tomography (CT) scan performed during hospitalization was evaluated. In cases where more than one X-ray was performed, only the first was analyzed. X-rays were classified as suggestive of immunosuppression if bilateral diffuse interstitial infiltrates were identified by two independent physicians. In cases of disagreement, a third physician reviewed the exam to provide a final assessment. CT findings were based on official reports prepared by a radiologist.

Laboratory analyses were conducted per clinical indication in accordance with institutional COVID-19 protocols. The initial complete blood count (CBC) was obtained within 24 h of admission. Parameters analyzed included RDW, leukocyte count and differential (neutrophils, lymphocytes), mean corpuscular volume (MCV), and platelet count. These variables were assessed both continuously and categorically (low/normal/high) based on the institutional reference range: 11.5% to 14.6%. RDW was additionally classified as either stable (remaining within normal limits throughout hospitalization) or elevated (exceeding the upper reference limit at any point during admission).

This study was conducted in accordance with the ethical principles set forth in the Declaration of Helsinki. The research protocol was reviewed and approved by the Institutional Review Board of UFMG (approval number 30437020.9.3001.5124). All participants were fully informed about the study's objectives, procedures, potential risks, and benefits, and provided written informed consent prior to their participation.

The data were analyzed by stratifying patients based on survival status. Continuous variables with a normal distribution were summarized using means and standard deviations, while categorical variables were presented as absolute numbers and percentages. A significance level of 5% (*p* < 0.05) was used to determine statistical significance. For data stratified into normal RDW and high RDW groups, the percentage of deaths within these groups was calculated and compared across two selected age categories (< 70 years and ≥ 70 years). The rationale for using 70 years as the age criterion was to investigate potential differences in RDW-related outcomes across age groups. This threshold is commonly employed in clinical studies to distinguish between younger and older populations, considering the physiological and clinical changes that occur with aging [8, 9]. Additionally, 72 years is recognized as the global life expectancy [8]. Other studies on COVID-19 mortality and comorbidities have shown that individuals aged 70 years or older are more likely to have underlying health conditions and, therefore, a higher risk of mortality [9].

Subsequently, the Gross and Mantel–Haenszel-adjusted risk ratios were calculated to assess the association between RDW levels and mortality risk. The same analytical procedure was applied to data stratified based on RDW upon admission (normal RDW upon admission vs. high RDW upon admission). A significance level of 5% (*p* < 0.05) was adopted for all statistical tests.

Next, for the data stratified into normal RDW and high RDW, the percentage of deaths in these groups was calculated, which was then compared within two selected age groups (< 70 years and 70 years or more), after which the Gross and Mantel–Haenszel-adjusted risk ratio was calculated. The same procedure was also used for data stratified into normal RDW upon admission and high RDW upon admission. The adopted significance level was 5% (*p* < 0.05). A time-to-death analysis was performed for variables that proved to be significant (*p* < 0.05) in the comparison between people who survived and died using the Cox proportional hazards model in univariate regression analysis. Those variables that revealed a significance level equal to or less than 20% (*p* < 0.20) formed the basis of an initial multivariate model, and only those with a significance level equal to or greater than 5% remained in this model (*p* < 0.05). Finally, Kaplan–Meier survival curves for the variables in the final model were estimated to obtain a better graphic visualization of the differences between the categories in time until death.

## 3. Results and Discussion

Of the 169 patients with laboratory-confirmed COVID-19 who were hospitalized during the study period, 161 were included in the analysis. Among them, 145 (90.1%) were admitted directly to respiratory isolation with a diagnosis of SARS, while 16 (9.9%) were admitted with nonspecific symptoms that led to suspicion of COVID-19 after an average of 6.6 days (range: 1 to 18 days). Eight patients with detectable RT-PCR results were excluded because they were suspected of having healthcare-associated COVID-19, indicating that they likely acquired the virus during their hospital stay.

The main characteristics presented by the patients included in the study are shown in Table 1. Of the 161 patients included, 39 (24.2%) died during hospitalization, 32 of them within the first 30 days, resulting in a 30-day mortality of 19.9%. Eighty patients (49.7%) were male, and 147 (91.3%) were self-declared black (black or brown). Mortality was 14.8% among women and 33.8% among men, with this difference being statistically significant (RR = 2.28; *p*=0.005). There was no difference in mortality rates between whites and blacks (42.7% vs. 22.5%; RR = 1.9; *p*=0.089). The mean age was 60.1 years (17 to 93, standard deviation - SD 16.9), with a median of 63 years. The mean age among survivors was 56.9 (16.7) years, while it was 70.3 (13.3) years among nonsurvivors, with a statistically significant difference (*p* < 0.001). In addition, patients over 70 years of age had a higher mortality rate (43.1% vs. 15.5%; RR = 2.78; *p* < 0.001). The mean time from symptom onset to hospital admission was 6.1 days (SD 4.6), with a median of 5 days. On average, patients had 2.44 comorbidities (SD 1.4), with the most common being systemic arterial hypertension (74 patients; 46%) and solid or hematologic malignancies (41 patients; 25.5%). Immunosuppression was identified in 71 patients (44.1%), including four patients living with HIV. Eight patients (5%) had no prior comorbidities, while 38 patients (23.6%) had one comorbidity, 45 (27.9%) had two, and 70 (43.5%) had three or more comorbidities.

Among the most reported comorbidities, chronic heart disease was found to be a significant risk factor for mortality (37.1% vs. 20.6%; RR = 1.8; *p*=0.044) in the univariate analysis. The presence of other comorbidities was not found to be significantly associated with mortality.

In this cohort, 62 patients (38.5%) developed a critical form of the disease, requiring admission to intensive care. Of these, 34 patients (54.8%) were admitted directly to the intensive care unit (ICU). The mean length of ICU stay was 12.9 days (SD 14.3). Invasive mechanical ventilation (MV) was required by 41 patients (25.5%), with a mean duration of 15.5 days (SD 19.3). The mortality rate was higher among patients who required intensive care (51.6% vs. 7.1%; RR = 7.26; *p* < 0.001) and those who required MV (73.2% vs. 7.3%; RR = 10.03; *p* < 0.001). However, there was no significant difference in mortality rates between patients admitted directly to intensive care and those initially admitted to the ward and later transferred to intensive care (47.1% vs. 57.1%; RR = 0.82; *p*=0.429). Furthermore, among those who required these interventions, there was no significant difference in the length of stay in intensive care between those who survived and those who did not (mean 13.6 days, SD 11.2 vs. mean 12.2 days, SD 17.3; *p*=0.7194). Similarly, the duration of MV did not differ significantly between survivors and nonsurvivors (mean 12.7 days, SD 10.6 vs. mean 23.1 days, SD 32.7; *p*=0.1275). A total of 144 patients (89.4%) underwent at least one chest X-ray during hospitalization. COVID-19 was deemed suggestive in 114 patients (79.2%). CT of the chest was performed in 110 (68.3%) patients, on average 9.3 days after the onset of symptoms (SD 5.7), with the results being compatible with COVID-19 in 81 cases (73.6%) in an official report prepared by a radiologist. The most common findings were as follows: ground-glass opacities in 91 cases (82.7%), which was diffuse with peripheral predominance in 81 cases (73.6%; 2 of these patients also had ground-glass opacities of bilateral central distribution) and localized in 10 cases (9.1%); septal thickening in 62 cases (56.4%); consolidation in 39 cases (35.5%); pleural effusion in 32 cases (29.1%); atelectasis in 28 cases (25.5%); lymphadenopathy in 23 cases (20.9%); and mosaic paving in 20 cases (18.2%). A chest X-ray considered suggestive of COVID-19 was associated with a higher mortality rate (30.7% vs. 10%; RR = 3.07; *p*=0.022). However, a chest CT that was reported as compatible with COVID-19 did not represent an additional risk of mortality (18.5% vs. 9.5%; RR = 1.95; *p*=0.512). Among the findings described in the CT scan reports, the presence of a mosaic paving pattern was found to be a risk factor for higher mortality (45% vs. 11.1%; RR = 4.05; *p* < 0.001). No difference was found in the time taken to perform a chest CT between survivors and deaths (mean 9.5, SD 5.7 vs. mean 8.4, SD 5.9; *p*=0.4478). Among the 52 patients who had at least one D-dimer measurement during their hospitalization, the average value was 2708 ng/mL (SD 4797). D-dimer levels were categorized as normal (reference < 500 ng/mL) in 7 patients (13.5%) and elevated in 45 patients (86.5%). No significant difference in D-dimer levels was observed between survivors and nonsurvivors, with mean values of 2518 (SD 4063) and 3418 (SD 7114), respectively (*p*=0.5857). Additionally, elevated D-dimer levels were not associated with increased mortality risk (22.2% vs. 14.3%; RR = 1.55; *p*=0.798).

The 161 patients included had at least one CBC performed during their hospital stay. A total of 2227 blood counts were conducted, with an average of 13.8 tests per patient (SD 12.9), ranging from 1 to 84 tests. Upon admission, the RDW was normal in 115 patients (71.4%), elevated in 45 (28%), and low in 1 (0.6%). The mean RDW value at admission was 14.5 (SD 2.4), which falls within the normal reference range. Of the patients with normal RDW at admission, 82 (71.3%) maintained normal levels throughout their stay, while 33 (28.7%) showed increased RDW levels over time. Among those with elevated RDW at admission, 40 (88.9%) remained elevated, while 5 (11.1%) returned to normal levels. The single patient with low RDW upon admission also returned to normal. When categorized, 88 patients (54.7%) maintained normal RDW, while 73 (45.3%) had elevated RDW throughout hospitalization. There was no significant difference in the mean RDW value at admission between survivors and nonsurvivors (14.4 [SD 2.4] for survivors vs. 14.9 [SD 2.4] for nonsurvivors; *p*=0.2081). The risk for mortality in the group with high RDW upon admission was higher than in the group with normal RDW, but without statistical significance (31.1% vs. 21.7%; RR = 1.43; *p*=0.413). However, in the subgroup of patients ≥ 70 years old, a statistical significance was observed in the mortality (66.7% for high RDW vs. 33.3% for normal RDW; RR = 2; *p*=0.029), Table 2. Furthermore, when subsequent laboratory findings of progression (RDW evolution, Table 1) are categorized, there is a higher risk of mortality among those with high RDW after admission (41.1% vs. 10.2%; RR = 4.03; *p* < 0.001). Moreover, when considering the RDW that changes at any time during hospitalization against such a normal parameter in all the blood count results, a significant risk of mortality tends to be observed for the entire cohort (39.7% vs. 9.6%; RR = 4.14; *p* < 0.001).

No significant difference was found in mortality between the different categories upon admission in relation to other parameters analyzed in the blood count, except for the absolute platelet counts between survivors and nonsurvivors (mean 216.3 × 10^3^, SD 110.7 vs. mean 171.2 × 10^3^, SD 80; *p*=0.0198).

Based on the Cox proportional hazards model (Table 3), the factors associated with lower survival were as follows: male gender (HR 2.3, 95% CI 1.1–4.5, *p*=0.018); age over 70 years (HR 3.3, 95% CI 1.7–6.5, *p* < 0.001); white race (HR 2.5, 95% CI 1.0–6.1, *p*=0.039); requirement for invasive MV (HR 4.9, 95% CI 2.2–10.6, *p* < 0.001); and the need for intensive care admission (HR 3.7, 95% CI 1.6–8.6, *p*=0.003).

**Figure 1:**
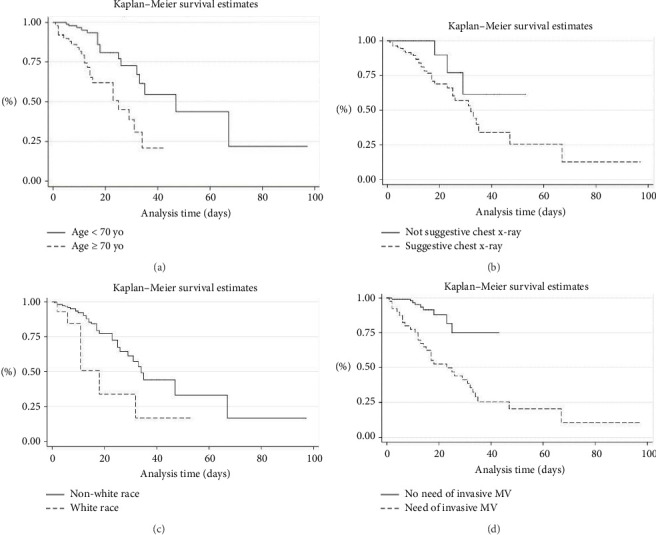
Kaplan–Meier curves for survival estimates of COVID-19-infected patients in a public university hospital in Belo Horizonte, Brazil, 2020. (a) Kaplan–Meier survival estimate by age: < 70 and ≥ 70 years. (b) Kaplan–Meier survival estimates by not suggestive chest X-ray and suggestive. (c) Kaplan–Meier survival estimates by non-white race and white race. (d) Kaplan–Meier survival estimates by non-need of invasive MV (mechanical ventilation) and need of MV.

The multivariate analysis identified the following as independent risk factors for lower survival: age > 70 years (HR 4.8, 95% CI 2.3–10.3, *p* < 0.001), white race (HR 3.2, 95% CI 1.2–8.61, *p*=0.018), and the need for invasive MV (HR 3.8, 95% CI 1.7–8.7, *p*=0.001). Additionally, a chest X-ray suggestive of COVID-19 was found to be a risk factor (HR 3.5, 95% CI 1.0–11.5, *p*=0.044).  Figure 1 presents the Kaplan–Meier curves for the variables associated with reduced survival in the multivariate analysis.

This study investigates mortality rates among patients with confirmed COVID-19 who were treated at a university tertiary hospital recognized as a referral center for complex oncohematological and transplantation cases. The aim was to identify the unique factors present in this patient population with various comorbidities, along with those contributing to higher disease severity and mortality. The overall mortality rate during hospitalization was 24.2%, with a 30-day mortality rate of 19.9%. Initial pandemic studies from 2020 reported a mortality rate of 15% in Wuhan to 17% in Boston among patients hospitalized with SARS [1, 6]. Our data indicate a higher mortality rate, likely due to the significant presence of oncological conditions as major comorbidities among our patient population. The data indicate a mean age of 60.1 years among patients hospitalized with SARS, with older age groups experiencing significantly higher mortality rates (43.1% in those aged 70 years or older, compared to 15.5% in younger patients; RR = 2.78; *p* < 0.001). Initial cohorts from China reported median ages ranging from 49 to 56 years among confirmed and hospitalized COVID-19 cases [1, 5]. However, later studies, such as a cohort study in Italy, found a higher median age of 65 years among patients with SARS [10]. Stratified by the same age categories, data from a multicenter study in New York reported similar mortality patterns, with an overall in-hospital mortality rate of 21%, and 42.83% for those aged 70 years or older compared to 9.97% in younger adults [11]. A Brazilian multicenter study, including patients from less complex hospitals, revealed a mortality rate of 35.88% among patients aged 65 years or older, in contrast to 12.88% among younger individuals [12].

Individuals in this study's cohort had an average of 2.44 comorbidities prior to admission. This is consistent with a subgroup of 355 patients from an Italian report, where the average number of pre-existing comorbidities in COVID-19 fatalities was 2.7 [10]. In our cohort, 95% of individuals had at least one comorbidity, with 23.6% having one, 27.9% having two, and 43.5% having three or more. These proportions closely resemble those reported in the Italian study [10]. The high prevalence of comorbidities observed in our cohort reflects the significant clinical complexity of patients treated in a high-complexity hospital, a trend observed in other studies. Notably, while conditions such as hypertension, obesity, and diabetes are commonly reported in other cohorts, our study identified a higher prevalence of more severe comorbidities, such as neoplasms, heart disease, and immunosuppression, all of which are critical factors for increased severity and mortality. This aligns with findings from the aforementioned meta-analysis, which included 42 studies and 423,117 patients from various countries, further highlighting the association between severe comorbidities and worse outcomes in COVID-19 [4].

Males corresponded to 49.7% of the cases, which is a risk factor for mortality (33.8% vs. 14.8%; RR = 2.28; *p*=0.005). Males also account for a disproportionately high number of critical cases and deaths in diverse cohorts worldwide [11, 13]. A Danish study showed a mortality rate among confirmed COVID-19 cases of 11.2% in males and 7.4% in females, with 47.1% of the individuals included being male [13]. A meta-analysis using data from 90 studies from 46 different countries totaling 3,111,714 individuals with a similar proportion of the sexes identified the male sex as a risk factor for 2.84 times more intensive care admissions and 1.39 times greater mortality [14].

The black race (self-declared black or brown individuals) represented more than 90% of this cohort, in contrast with the proportions found in the Brazilian population, where in the 2019 National Household Sample Survey (in Portuguese: Pesquisa Nacional por Amostra de Domicílios—PNAD), 42.7% of Brazilians declared themselves as white, 46.8% as brown, 9.4% as black, and 1.1% as yellow or indigenous (https://painel.ibge.gov.br/pnadc/).

This percentage may reflect disparities in social determinants of health affecting this patient group in Brazil, consistent with findings from a large study in England. In that study, even after adjusting for multiple factors, Black and South Asian individuals were at higher risk than their White counterparts (HR 1.48, 95% CI 1.29–1.69 and HR 1.45, 95% CI 1.32–1.58, respectively) [6]. There was a nonsignificant trend toward higher mortality in White patients, and these patients also showed lower survival in the Cox model. However, the small proportion of self-identified White patients makes it difficult to generalize these findings in our study. In an American study conducted in Wisconsin, both race and poverty were linked to an increased risk of hospitalization among individuals with COVID-19, but only poverty was associated with an increased risk of ICU admission [15].

A median of 5 days of symptoms at the time of hospitalization was slightly lower than that found in other studies [1, 6]. One Chinese study showed a median of 5 days for the onset of dyspnea and 7 days for hospital admission [6]. Another also Chinese study found a median of 8 days for the onset of dyspnea [1].

This study's data also draws attention to one of the difficulties in the management of respiratory syndromes: the etiological diagnosis. Despite the collection being done shortly after admission, the test results of swab exams take an average of 3 days to be released. One study showed a mean time of 15.4 h for RT-PCR results [11]. The delay in making the etiological diagnosis of pulmonary conditions can lead to the unnecessary use of antimicrobials and their consequences, as well as the need to use other more invasive forms of workup to clarify the diagnosis [16].

In this cohort, 38% of hospitalized patients required intensive care, which aligns with ranges reported in other studies. For instance, in Petrilli et al. [17], 65.4% of patients required MV, while 10.3% required intensive care without ventilation. In our study, 25.5% needed MV, and their mortality rate reached 73%, higher than the 60% observed in the New York cohort [17].

The severity of underlying health conditions likely contributed to this outcome, as patients were treated in a high-complexity hospital. Although not statistically significant, there was a trend toward longer MV among survivors, suggesting that early death may be associated with shorter ventilation duration. Chest X-rays, being simple, quick, and cost-effective, played a crucial role in diagnosing suspected COVID-19 cases, with 79.2% of patients showing suggestive findings. Additionally, they proved valuable in determining patient outcomes. The fact that roughly 20% of chest X-rays showed no abnormalities is consistent with existing literature, such as Wong et al., who reported that 69% of patients had abnormal baseline chest radiography findings [18]. The availability of such a readily accessible test, which can predict disease severity, allows for prompt initiation of appropriate treatment strategies.

The most frequent findings on chest CT scans in this cohort were consistent with other studies, notably ground-glass opacities, and septal thickening. Consolidations were somewhat less common, while pleural thickening and air bronchograms were infrequently observed. In contrast, unlike other imaging studies [18], which observed a low incidence of pleural effusion (3%), our patients exhibited a higher frequency of pleural effusion and atelectasis, possibly associated with the presence of additional comorbidities.

While other studies have demonstrated that elevated D-dimer levels reflect the procoagulant changes seen in COVID-19, such as Liao et al. in China, higher D-dimer levels (> 2 mg/L) were significantly associated with mortality (OR 4.41, 95% CI 1.06–18.30; *p*=0.041) [19, 20]. This finding was not observed in our study. A likely reason is the small number of patients who underwent D-dimer testing. Notably, in our cohort, D-dimer was measured primarily when there was clinical suspicion of pulmonary thromboembolism or deep venous thrombosis, rather than as a routine evaluation of hypercoagulability in COVID-19.

The presence of lymphopenia was a common finding in most patients (58.4%). It was also common in other studies, despite the variation in the total leukocyte count [21, 22]. A meta-analysis demonstrated that patients with severe disease, including those admitted to the ICU or suffering from acute respiratory distress syndrome, often had significantly lower lymphocyte counts compared to nonsevere cases. This correlation is particularly evident in patients under 55 years old, where lymphopenia is strongly associated with a poorer prognosis [22]. Despite its clear association with severe disease, lymphopenia is not necessarily a direct cause of poor outcomes but rather a marker of the body's overwhelmed immune response, which underscores its utility in clinical settings for predicting disease severity and informing treatment plans [23].

A high RDW upon admission was not statistically significant as a risk factor for mortality in this cohort (31.1% vs. 21.7%; RR = 1.43; *p*=0.413). However, this finding was associated with higher mortality in the subgroup of patients aged 70 years and over (66.7% vs. 33.3%; RR = 2; *p*=0.029). Furthermore, a high RDW during hospitalization after admission was a factor for mortality in the entire cohort (41.1% vs. 10.2%; RR = 4.03; *p* < 0.001), and if all measurements are considered (admission plus elevation), having a high RDW at any time during the hospital stay was associated with higher mortality (39.7% vs. 9.6%; RR = 4.14; *p* < 0.001).

Some studies have linked elevated RDW values to severe disease and higher mortality in COVID-19. A critical review of the literature, focusing on the prognostic role of RDW, found that the absolute RDW-CV was 0.69% higher (95% CI 0.40%–0.98%; *p* < 0.001) in patients with severe illness compared to those with milder forms of the disease [24].

This may explain why our study found an association between RDW and mortality when age was stratified at such a high age (70 and over), where critical forms may occur more frequently, but not over the entire spectrum of the survival curve. In other words, the association was only true for higher ages. Prior studies have consistently demonstrated that RDW increases progressively with age and is strongly associated with mortality in community-dwelling older adults [25]. This age-related variability in RDW may partially explain why the association between RDW and mortality was observed in our ≥ 70-year subgroup but not in the overall cohort. Furthermore, it is important to note that the observation of RDW being predictive of mortality in patients aged ≥ 70 years, but not in the overall cohort, should be interpreted cautiously. This finding may reflect limited statistical power in subgroup analyses or the presence of unmeasured confounding factors that were not fully accounted for in our models. However, consistent with our observations, Lucijanić et al. [26] identified RDW as an important component of a prognostic score, together with advanced age, confirming that in patients over 70 years, RDW remains a significant marker of mortality risk. We recognize this as a limitation that may have influenced the results, and we emphasize that this association should be considered hypothesis-generating.

A study involving 1641 patients found a relative risk of mortality of 2.73 in patients with elevated RDW at admission compared to those with normal RDW at the same time point [6]. A meta-analysis of 10 studies that included 14,866 patients linked higher RDW levels to worse outcomes when comparing cases (death or severe COVID-19 symptoms) and controls (survivors or mild symptoms). It reported a mean difference of 0.72 (95% CI 0.47–0.97; *I*^2^=89.51%), with a difference of 0.93 between deaths and survivors (95% CI 0.63–1.23; *I*^2^=85.58%) and 0.61 between patients with severe and mild symptoms (95% CI 0.28–0.94; *I*^2^=82.18%) [21].

Factors that may explain the time-dependent association with mortality, without achieving statistical significance at admission, include the relatively small number of patients in our study and the high proportion of individuals with neoplasms, who are known to have an increased likelihood of an altered RDW value [6, 27]. Furthermore, we hypothesize that patients with multiple pre-existing comorbidities might experience earlier decompensation evidenced by a shorter duration of symptoms upon admission, while we still emphasize the importance of dynamic changes in biomarkers for predicting mortality. Some studies support this perspective. One study identified time-dependent differences in hematologic parameters between survivors and nonsurvivors: the mean neutrophil count, although initially similar (*p*=0.191), began to diverge from the sixth day of symptoms, remaining within normal ranges for survivors but rising markedly in nonsurvivors (*p* < 0.001). The lymphocyte count was lower in nonsurvivors (*p* < 0.001) and already differed from survivors on the first day after symptom onset (mean difference 330.1; 95% CI 633.48–26.85; *p*=0.033), a gap that widened over time (765.73; 95% CI 1050.04–481.43; *p* < 0.001). The red blood cell count was also lower in nonsurvivors (*p* < 0.001), initially similar at the onset of symptoms (*p*=0.257) but diverging after 2 days (*p*=0.033), eventually dropping below 3.8 million cells/mm^3^ by Day 7, while survivors maintained normal values. Hemoglobin levels were comparable between groups at the onset of symptoms (*p*=0.063) but began to differ the following day (*p*=0.041), remaining lower in nonsurvivors. MCV was higher in nonsurvivors (*p* < 0.001), similar at the start (*p*=0.066), but became different on the next day (*p*=0.024), though still within reference ranges for both groups. RDW was higher among nonsurvivors from the onset (*p*=0.004), displaying a slight increase in this group over time but staying within normal limits for survivors (*p* < 0.001) [28]. This observation of evolving differences is consistent with acute inflammation followed by either recovery (i.e., survival) or death, underscoring the need to consider these parameters over the course of the disease [28]. Moreover, the slightly elevated mean RDW values at admission reported in other studies, just above the normal cutoff of 14.5%, were 14.6% (IQR, 13.7–16.3) and 14.8% (SD 3.0) [29]. Those studies included a larger number of patients, potentially highlighting the biomarker's importance as early as hospital admission. However, the median of 5 days of symptoms at the time of admission in our study, in addition to reflecting earlier hospitalization, may explain the later emergence of these abnormalities during hospitalization, corresponding to the anemia and anisocytosis observed in later stages of COVID-19.

Another study from Massachusetts General Hospital corroborated these findings, showing that an RDW greater than 14.5% at admission was linked to a significantly higher mortality risk, which remained robust even after adjusting for various demographic and clinical factors. These insights underline the utility of RDW as a biomarker for risk stratification and prognosis in COVID-19 [30].

Although some studies have found a correlation between RDW elevation and mortality, recognizing RDW as a simple, fast, and inexpensive biomarker, our study did not confirm this association in the final statistical models.

The lack of association found in our study may be attributed to the immunosuppressive and cancer characteristics of our patients, the small sample size, or other design limitations. Future prospective and multicentric studies should consider using a combination of risk factors, such as age and RDW, to better predict negative outcomes in COVID-19 in Brazilian patients. Given that a recent Dutch intensive care study found increased RDW at ICU admission in COVID-19 patients to be associated with higher 30-day and 90-day mortality rates, as well as shorter durations of invasive MV [31], a focused analysis on patients admitted to the ICU could offer more targeted insights.

Other studies in Brazil have examined hematologic parameters related to COVID-19, often focusing on noncritical or nonsolely hospitalized patients. In these studies, confounding factors from other infectious diseases, such as dengue or other viral infections, may have also contributed to hematologic alterations [32].

Another recent Brazilian cross-sectional study conducted in Mato Grosso, involving 251 patients, found that RDW and other hematological parameters were significantly associated with mortality during both COVID-19 waves (May 2020 and August 2021) [33].

Our study is a single center with a clinical profile and comorbidities that differ from those found in other general hospitals or clinics that do not manage complex cases, as our university hospital is a reference for such cases. The large proportion of immunosuppressed, cancer, and transplant patients in our cohort likely had altered RDW values even before SARS-CoV-2 infection, which could have influenced our findings. Additionally, various underlying acute and chronic conditions can affect RDW, making it a potential marker for comorbidities that contribute to unfavorable outcomes in COVID-19. Nonetheless, although the study design is robust, the interpretation that RDW was not an independent predictor of mortality should be viewed in light of certain limitations, including the relatively small sample size, the high frequency of underlying conditions that could independently influence RDW values, and the limited racial diversity in our cohort, factors that may restrict the broader applicability of these findings. Furthermore, it is important to note that the observation of RDW being predictive of mortality in patients aged ≥ 70 years, but not in the overall cohort, should be interpreted cautiously. This finding may reflect limited statistical power in subgroup analyses or the presence of unmeasured confounding factors that were not fully accounted for in our models.

We are drafting this journal paper following the release of its preprint version, which allowed us to share our initial findings with the scientific community. Since then, we have made substantial revisions, including rewriting and reinterpreting certain data. This final manuscript incorporates numerous refinements, further enhancing clarity and precision while correcting some interpretations presented in the preprint [34].

## 4. Conclusions

In our cohort of hospitalized COVID-19-infected patients, RDW at admission did not emerge as a statistically significant predictor of mortality. Despite the initial observation that patients with elevated RDW had a higher risk of mortality compared to those with normal RDW (31.1% vs. 21.7%), this association lacked statistical significance (*p*=0.413). Additionally, no significant differences in mean RDW values were observed between survivors and nonsurvivors (14.4 vs. 14.9; *p*=0.2081).

While RDW has been identified as a potential prognostic marker in various disease contexts, our findings suggest that, in this population, RDW may not be a reliable independent predictor of outcomes in COVID-19-infected patients. The multivariate analysis highlighted other factors, such as advanced age, white race, and the need for invasive MV or a chest X-ray suggestive of COVID-19, as stronger predictors of mortality.

These results underscore the complexity of identifying reliable biomarkers for predicting outcomes in COVID-19. Future studies with larger sample sizes and diverse populations are warranted to further explore the role of RDW and other hematologic parameters in the prognosis of COVID-19-infected patients.

## Figures and Tables

**Table 1:** Baseline characteristics, comorbidities, laboratory, and radiological findings of COVID-19-infected patients, survivors, and nonsurvivors, in Belo Horizonte, Brazil, 2020.

Features		Total *N* = 161 (100.0%)	Survivor *N* = 122 (75.8%)	Death *N* = 39 (24.2%)	*p* value
Age	Mean (SD)	60.1 (16.9)	56.9 (16.7)	70.3 (13.3)	< 0.001
Age ≥ 70 years	Yes	51 (31.7)	29 (56.9)	22 (43.1)	0.000
Sex	Male	80 (49.7)	53 (66.3)	27 (33.8)	0.005
Race/skin color	Black	147 (91.3)	114 (77.6)	33 (22.5)	0.089
Comorbidities	Mean (SD)	2.44 (1.4)	2.42 (1.4)	2.5 (1.5)	0.7184
BMI > 30 kg/m^2^	Yes	20 (12.4)	18 (90)	2 (10)	0.163
Previous smoking	Yes	34 (21.1)	26 (76.5)	8 (23.5)	0.916
Current smoking	Yes	8 (5.0)	4 (50)	4 (50)	0.098
Systemic arterial hypertension	Yes	74 (46.0)	54 (73)	20 (27)	0.444
Solid or hematological neoplasm	Yes	41 (25.5)	29 (70.7)	12 (29.3)	0.383
Diabetes mellitus	Yes	38 (23.6)	27 (71.1)	11 (29)	0.437
Chronic heart disease	Yes	35 (21.7)	22 (62.9)	13 (37.1)	0.044
Chronic lung disease	Yes	26 (16.1)	19 (73.1)	7 (26.9)	0.726
Chronic kidney disease	Yes	18 (11.2)	14 (77.8)	4 (22.2)	1.000
Solid organ transplant	Yes	12 (7.5)	10 (83.3)	2 (16.7)	0.732
Immunosuppression	Yes	71 (44.1)	52 (73.2)	19 (26.8)	0.505
Time of symptoms upon admission	Mean (SD)	6.07 (4.6)	6.4 (4.7)	5.2 (4.0)	0.1528
Time of symptoms until swab collection^1^		8.0 (5.3)	8.3 (5.2)	7.3 (5.6)	0.3006
Time of hospitalization until swab collection^1^		2.0 (4.0)	2.0 (3.8)	2.1 (4.5)	0.8162
Time until swab result^1^		3.1 (1.7)	3.1 (1.7)	3.4 (1.5)	0.2941
Hospitalization time^2^		16.0 (12.9)	15.4 (12.7)	17.8 (13.6)	0.3185
Need for intensive care	Yes	62 (38.5)	30 (48.4)	32 (51.6)	0.000
Direct admission to ICU	Yes	34 (54.8)	18 (52.9)	16 (47.1)	0.429
ICU time	Mean (SD)	12.9 (14.3)	12.2 (17.3)	13.6 (11.2)	0.7194
Need for invasive mechanical ventilation	Yes	41 (25.5)	11 (26.8)	30 (73.2)	0.000
Invasive mechanical ventilation time	Mean (SD)	15.5 (19.3)	23.1 (32.7)	12.7 (10.6)	0.1275
Suggestive chest X-ray^3^	Yes	114 (79.2)	79 (69.3)	35 (30.7)	0.022
Compatible chest CT^4^	Indeterminate	8 (7.3)	6 (75)	2 (25)	0.512
	No	21 (19.1)	19 (90.5)	2 (9.5)	
	Yes	81 (73.6)	66 (81.5)	15 (18.5)	

Time of symptoms until CT	Mean (DP)	9.3 (5.7)	9.5 (5.7)	8.4 (5.9)	0.4478
Ground glass	Yes	91 (82.7)	73 (80.2)	18 (19.8)	0.186
Peripheral diffuse ground glass	Yes	81 (73.6)	64 (79)	17 (21)	0.149
Central diffused ground glass	Yes	2 (1.8)	2 (100)	0 (0.0)	1.000
Localized ground glass	Yes	10 (9.1)	9 (90)	1 (10)	1.000
Consolidation	Yes	39 (35.5)	29 (74.4)	10 (25.6)	0.085
Septal thickening	Yes	62 (56.4)	49 (79)	13 (21)	0.244
Bronchial thickening	Yes	7 (6.4)	7 (100)	0 (0)	0.602
Pleural thickening	Yes	4 (3.6)	2 (50)	2 (50)	0.137
Mosaic paving	Yes	20 (18.2)	11 (55)	9 (45)	0.000
Atelectasis	Yes	28 (25.5)	22 (78.6)	6 (21.4)	0.500
Pleural effusion	Yes	32 (29.1)	26 (81.3)	6 (18.8)	0.793
Lymphadenopathy	Yes	23 (20.9)	19 (82.6)	4 (17.4)	1.000
Air bronchogram	Yes	3 (2.7)	2 (66.7)	1 (33.3)	0.437
Pericardial effusion	Yes	9 (8.2)	8 (88.9)	1 (11.1)	1.000
Cardiomegaly	Yes	17 (15.5)	14 (82.4)	3 (17.7)	1.000
Nodule(s)	Yes	16 (14.5)	11 (68.8)	5 (31.3)	0.110
Micronodule(s)	Yes	12 (10.9)	10 (83.3)	2 (16.7)	1.000
Mass(es)	Yes	3 (2.7)	1 (33.3)	2 (66.7)	0.077
Pulmonary hypertension	Yes	12 (10.9)	10 (83.3)	2 (16.7)	1.000
Tree-in-bud	Yes	1 (0.9)	0 (0)	1 (100)	0.173
Bronchiectasis	Yes	6 (5.5)	6 (100)	0 (0)	0.587
Pulmonary infarction	Yes	3 (2.7)	2 (66.7)	1 (33.3)	0.437
Emphysema	Yes	9 (8.2)	7 (77.8)	2 (22.2)	0.652
Number of recovered blood counts	Mean (SD)	13.8 (12.9)	12.8 (12.9)	17 (12.5)	0.0778
RDW upon admission		14.5 (2.4)	14.4 (2.4)	14.9 (2.4)	0.2081
Leukocyte count upon admission		9.84 (25.0)	9.9 (28.5)	9.5 (6.6)	0.9320
Lymphocyte count upon admission		1.30 (2.0)	1.38 (2.1)	1.02 (1.1)	0.3374
Neutrophil count upon admission		6.20 (6.7)	5.9 (7.32)	7.0 (4.4)	0.4079
Platelet count upon admission		205.3 (105.6)	216.3 (110.7)	171.2 (80.0)	0.0198
VCM upon admission		86.6 (7.3)	86.6 (7.3)	86.6 (7.3)	0.9973
First D-dimer performed upon admission^5^		2708 (4797)	2518 (4063)	3418 (7114)	0.5857
RDW category	High	45 (28.0)	31 (68.9)	14 (31.1)	0.413
	Normal	115 (71.4)	90 (78.3)	25 (21.7)	
	Low	1 (0.6)	1 (100)	0 (0.0)	

RDW evolution	High	73 (45.3)	43 (58.9)	30 (41.1)	0.000
	Normal	88 (54.7)	79 (89.8)	9 (10.2)	

Leukocyte category	High	46 (28.6)	40 (87)	6 (13)	0.094
	Normal	94 (58.4)	66 (70.2)	28 (29.8)	
	Low	21 (13)	16 (76.2)	5 (23.8)	

Lymphocyte category	High	6 (3.7)	4 (66.7)	2 (33.3)	0.301
	Normal	61 (37.9)	50 (82)	11 (18)	
	Low	94 (58.4)	68 (72.3)	26 (27.7)	

Neutrophil category	High	108 (67.1)	77 (71.3)	31 (28.7)	0.197
	Normal	40 (24.8)	34 (85)	6 (15)	
	Low	13 (8.1)	11 (84.6)	2 (15.4)	

Platelet category	High	20 (12.4)	18 (90)	2 (10)	0.187
	Normal	110 (68.3)	83 (75.5)	27 (24.6)	
	Low	31 (19.3)	21 (67.7)	10 (32.3)	

VCM category	High	11 (6.8)	7 (63.6)	4 (36.4)	0.631
	Normal	116 (72.1)	89 (76.7)	27 (23.3)	
	Low	34 (21.1)	26 (76.5)	8 (23.5)	

D-dimer category^5^	High	45 (28.0)	35 (77.8)	10 (22.2)	0.798
	Na	109 (67.7)	81 (74.3)	28 (25.7)	
	Normal	7 (4.3)	6 (85.7)	1 (14.3)	

^1^4 patients underwent external swab before admission. For this variable: total 157, survivors 118, deaths 39.

^2^1 patients remained hospitalized at the end of follow-up. For this variable: total 160, survivors 121, deaths 39.

^3^144 patients underwent chest X-ray. For this variable: total 144, survivors 106, deaths 38.

^4^110 patients underwent chest CT. For this variable: total 110, survivors 91, deaths 19.

^5^52 patients had D-dimer levels registered. For this variable: total 52, survivors 41, deaths 11.

**Table 2:** Mortality risk according to age-stratified RDW category (normal or high) of COVID-19-infected patients, in Belo Horizonte, Brazil, 2020.

Age group	Normal RDW during hospitalization		High RDW during hospitalization		*p* value	Risk ratio (95% CI)
	*N*	Mortality [*n* (%)]	*N*	Mortality [*n* (%)]		
< 70	59	4 (6.8)	51	13 (25.5)	0.007	3.76 (1.31; 10.8)
≥ 70	24	4 (16.7)	27	18 (66.7)	< 0.001	4.00 (1.58; 10.2)
Total cohort	83	8 (9.6)	78	31 (39.7)	< 0.001	

Gross						4.12 (2.02; 8.41)
Mantel–Haenszel method						3.89 (1.93; 7.83)

**Age group**	**Normal RDW at admission**		**High RDW at admission**		**p** **value**	**Risk ratio (95% CI)**
	***N***	**Mortality [*n* (%)]**	***N***	**Mortality [*n* (%)]**		

< 70	80	13 (16.3)	30	4 (13.3)	> 0.999	0.82 (0.29; 2.31)
> 70	36	12 (33.3)	15	10 (66.7)	0.029	2.00 (1.07; 3.74)
Total cohort	116	25 (21.6)	45	14 (31.1)	0.204	

Gross						1.44 (0.82; 2.55)
Mantel–Haenszel method						1.41 (0.83; 2.39)

**Table 3:** Cox model results and survival analysis of baseline characteristics, comorbidities, laboratory, and radiological findings of COVID-19-infected patients, survivors, and nonsurvivors, in Belo Horizonte, Brazil, 2020.

Variable	Univariate		Multivariate	
	HR (95% CI)	*p* value	HR (95% CI)	*p* value
Sex				
Female	1.00	—	—	—
Male	2.3 (1.1; 4.5)	0.018	—	—
Age (in years)				
< 70	1.0	—	1.00	—
≥ 70	3.3 (1.7; 6.5)	< 0.001	4.8 (2.3; 10.3)	< 0.001
Race/color				
Not white	1.0	—	1.00	—
White	2.5 (1.0; 6.1)	0.039	3.2 (1.2; 8.6)	0.018
Suggestive chest X-ray				
No	1.0	—	1.00	—
Yes	3.2 (1.0; 10.5)	0.052	3.5 (1.0; 11.5)	0.044
Compatible chest CT				
No	1.0	—	—	—
Yes	1.6 (0.7; 3.6)	0.218	—	—
Solid or hematological neoplasm				
No	1.0	—	—	—
Yes	1.8 (0.9; 3.7)	0.095	—	—
Chronic heart disease				
No	1.0	—	—	—
Yes	1.9 (1.0; 3.8)	0.053	—	—
Chronic lung disease				
No	1.0	—	—	—
Yes	1.3 (0.6; 2.9)	0.584	—	—
Immunosuppression				
No	1.0	—	—	—
Yes	1.6 (08; 3.0)	0.173	—	—
Leukocyte category				
Low	1.1 (0.4; 2.8)	0.892	—	—
Normal	1.0	—	—	—
High	0.5 (0.2; 1.1)	0.091	—	—
Neutrophil category				
Low	0.7 (0.1; 3.5)	0.647	—	—
Normal	1.0	—	—	—
High	1.6 (0.7; 3.9)	0.287	—	—
VCM category				
Low	0.8 (0.4; 1.9)	0.670	—	—
Normal	1.0	—	—	—
High	1.4 (0.5; 3.9)	0.559	—	—
Need for invasive MV				
No	1.0	—	1.00	—
Yes	4.9 (2.2; 10.6)	< 0.001	3.8 (1.7; 8.7)	0.001
Need for ICU				
No	1.0	—	—	—
Yes	3.7 (1.6; 8.6)	0.003	—	—
High RDW while hospitalized (> 14.6%)				
No	1.0	—	—	—
Yes	1.9 (0.9; 4.1)	0.102	—	—
High RDW upon admission or while hospitalized				
No	1.0	—	—	—
Yes	1.9 (0.8; 4.2)	0.133	—	—

## Data Availability

The data from this clinical study in the field of medicine are available upon request to the authors, subject to prior evaluation.
